# Nutrients Associated with Sleep Bruxism

**DOI:** 10.3390/jcm12072623

**Published:** 2023-03-31

**Authors:** Naoki Toyama, Daisuke Ekuni, Daiki Fukuhara, Nanami Sawada, Miho Yamashita, Momoe Komiyama, Takahiko Nagahama, Manabu Morita

**Affiliations:** 1 Department of Preventive Dentistry, Academic Field of Medicine, Dentistry and Pharmaceutical Sciences, Okayama University, Okayama 700-8558, Japan; 2 Department of Preventive Dentistry, Okayama University Hospital, Okayama 700-8558, Japan; 3 Department of Foods and Human Nutrition, Notre Dame Seishin University, Okayama 700-0013, Japan

**Keywords:** sleep bruxism, dietary fiber, electromyography, young adult, biostatistics, nutrition assessment

## Abstract

Background: The purpose of the present research was to identify nutrients related to sleep bruxism and to establish a hypothesis regarding the relationship between sleep bruxism and nutrients. Methods: We recruited 143 Japanese university students in 2021 and assigned them to sleep bruxism (*n* = 58) and non-sleep bruxism groups (*n* = 85), using an identical single-channel wearable electromyography device. To investigate nutrient intakes, participants answered a food frequency questionnaire based on food groups. We assessed differences in nutrient intakes between the sleep bruxism and non-sleep bruxism groups. Results: Logistic regression modeling showed that sleep bruxism tended to be associated with dietary fiber (odds ratio, 0.91; 95% confidence interval, 0.83–1.00; *p* = 0.059). In addition, a subgroup analysis selecting students in the top and bottom quartiles of dietary fiber intake showed that students with sleep bruxism had a significantly lower dietary fiber intake (10.4 ± 4.6 g) than those without sleep bruxism (13.4 ± 6.1 g; *p* = 0.022). Conclusion: The present research showed that dietary fiber intake may be related to sleep bruxism. Therefore, we hypothesized that dietary fiber would improve sleep bruxism in young adults.

## 1. Introduction

Sleep bruxism (SB) is defined by the American Academy of Sleep Medicine as “repetitive jaw muscle activity characterized by the clenching or grinding of teeth or bracing or thrusting of the mandible”. SB causes considerable bite force, with a reported mean of 42.3 kgf (range, 15.6–81.2 kgf) in Japanese young adults [1]. The force generated during SB can harm the masticatory system, particularly the teeth, periodontium, and articulations of the mandible with the skull (temporomandibular joint disorder) [2]. Since no definitive therapy has been established, clarification of the pathophysiology of SB is important.

The etiology of SB is related to the activities of the central and autonomic nervous systems during sleep. The sleep cycle is composed of non-rapid eye movement (NREM) sleep (stages 1–4 in Rechtschaffen and Kales criteria, also known as stages N1–N3 in 2007 AASM scoring manual) and rapid eye movement (REM) sleep. The natural sleep cycle starts with the deepening of NREM sleep (stages 1 and 2; stages N1 and N2), reaches deep NREM (stages 3 and 4; stage N3), then shifts toward light NREM and REM sleep [3]. SB is followed by micro-arousal, representing the temporary activity of central and sympathetic nerves [4]. SB and micro-arousal are highest during NREM stages 1 and 2 [5,6]. Slow-wave sleep (SWS), an index of deep sleep, decreases following SB onset [5]. A shift in the sympathovagal balance towards increased sympathetic activity starts from around 8 min before SB onset [5]. This suggests that SB is related with low sleep quality [7]. In addition, a previous study showed that SB was associated with certain SNPs, sleep disordered breathing, and other factors such as sexomnia [8,9,10,11].

Nutrients play an important role in the regulation of sleep wellness [12]. For example, foods and meals that contain sufficient protein, carbohydrates, and fats are essential for maintaining the quality of sleep [13]. The amino acid tryptophan, the precursor of melatonin, has a positive effect on sleep [14]. Carbohydrate-containing meals with a low glycemic index, low glycemic load, and high fiber content can improve sleep quality [15]. Caffeine causes a decrease in total amount and quality of sleep, and delays sleep induction [16]. Carbohydrates, lipids, amino acids, and vitamins are related to sleep disorders [17]. In this way, a growing body of evidence demonstrates that nutrition can significantly influence sleep. However, whether nutrients can influence SB remains unclear. Since the relationship between nutrients and sleep is complicated [17], nutrients related to SB need to be identified, before performing confirmatory (evidential) research. The purpose of the present exploratory research was to identify nutrients related to SB and to establish a hypothesis regarding the relationship between SB and nutrients.

## 2. Materials and Methods

### 2.1. Study Population

The present cross-sectional study recruited students belonging to Okayama University or the Department of Food and Nutrition at Notre Dame Seishin University from April 2021 to August 2022. Students who underwent an oral examination and completed self-reported questionnaires were included. Those who declined to participate or who reported reflux esophagitis or regularly taking selective serotonin reuptake inhibitors were excluded [18,19]. We estimated that the minimum sample size required 57 students in the SB group and 75 students in the non-sleep bruxism (NSB) group, based on α = 0.05, power (1 − β) = 0.8, effect size = 0.5, and allocation ratio (SB group/NSB group) = 0.75 for the two-tailed *t* test.

### 2.2. Ethical Procedures and Informed Consent

The present study was approved by the ethics committees of Okayama University Graduate School of Medicine, Dentistry and Pharmaceutical Sciences and Okayama University Hospital (approval no. 2104-017), as well as the Ethics Committee of Notre Dame Seishin University, and was conducted in accordance with the principles of the Declaration of Helsinki. All participants provided written informed consent to participate in the study. The study was conducted and reported in accordance with STROBE (strengthening the reporting of observational studies in epidemiology) guidelines.

### 2.3. Assessment of Risk Factors for SB and of Subjective Sleep Quality

We investigated sex, age, underlying systemic diseases, medicines being taken regularly, and smoking status using self-reported questionnaires (Supplementary File S1). For the assessment of subjective sleep quality, we used the Japanese version of the Pittsburg sleep quality index (PSQI-J) [20]. The questionnaires were answered using Google Form or printed out and filled in by respondents.

### 2.4. Screening of the SB and NSB Groups

**Figure 1:**
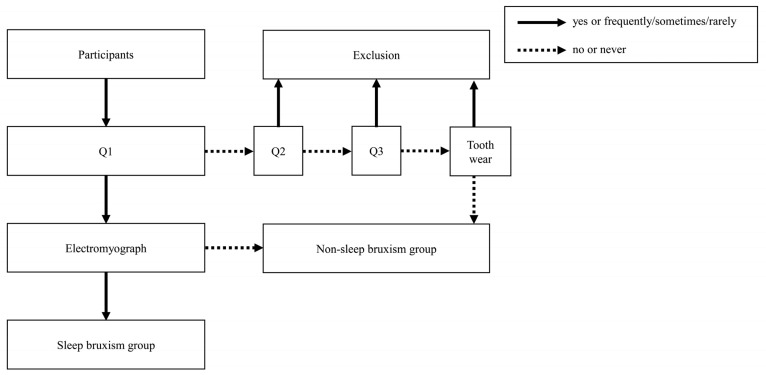
Protocol for assignment to the sleep bruxism and non-sleep bruxism groups.

Figure 1 shows the protocol for assignment to the SB and the NSB groups. Students underwent oral examination and completed self-reported questionnaires. Seven dentists, who were calibrated before the oral examinations (kappa value >0.8), clinically checked for abnormal tooth wear with exposed dentin at the canine or first or second premolars. In addition, we asked the following three questions to screen for SB [21]:

During the past 3 months,

Q1: Has anyone heard you grinding your teeth while you sleep at night? (yes/no);
Q2: How often does your jaw feel fatigued or sore on waking in the morning? (frequently/sometimes/rarely/never);
Q3: How often do you experience headache in the temples on waking in the morning? (frequently/sometimes/rarely/never).

Students who did not show abnormal tooth wear in the oral examination and answered “no” or “never” to each of Q1–3 were assigned to the NSB group, since these questions are highly specific [22]. In addition, students who answered “yes” to Q1 were assessed for SB using an identical single-channel wearable electromyography (EMG) device (EMG Logger; GC Co., Tokyo, Japan).

### 2.5. Diagnosis of SB

After recording myoelectrical data from the masticatory muscles, we analyzed the results using W-EMG Viewer (GC Co.). We identified peak bite forces over 10% of the maximal voluntary contraction (MVC) and under 300% MVC as burst waves. Episodes were then decided as follows [23]:

1. Phasic episode: burst occurred ≥3 times, and each burst continued ≥0.25 s but <2 s.
2. Tonic episode: a burst continued ≥2 s.
3. Mixed episode: both phasic and tonic episodes were measured.

We then assessed whether the results met any of the following diagnostic criteria [23]:

1. Total number of episodes (phasic, tonic and mixed) >30 times/night.
2. Number of episodes (phasic, tonic or mixed) >4 episodes/h.
3. Number of bursts >6 bursts/episode.
4. Number of bursts >25 bursts/h.

Students who met the diagnostic criteria were assigned to the SB group, and those who did not meet these criteria were assigned to the NSB group.

### 2.6. Assessment of Nutrients

To assess nutrient intake, we used the food frequency questionnaire based on food groups (FFQg) version 6 (Kenpakusha Co., Tokyo, Japan) and Excel Eiyo-kun version 9 (Kenpakusha Co.). This general assessment tool for nutrient intake in Japan has shown high reproducibility and reasonable validity [24]. Participants recorded the frequency of intake of 30 food groups and 10 types of cooking in the past month. To allow participants to better gauge food intake, we used food samples to reduce errors (Basic 90 Food model for nutrition guidance; IWASAKI Co., Osaka, Japan).

We calculated nutrient intake based on dietary reference intakes for Japanese (2020) [25]. Nutrients investigated included water (g), cholesterol (mg), monounsaturated fatty acids (g), polyunsaturated fatty acids (g), energy (kcal), protein (g), lipids (g), saturated fatty acids (g), n-3 fatty acids (g), n-6 fatty acids (g), carbohydrate (g), dietary fiber (g), vitamin A (μg), vitamin D (μg), vitamin E (mg), vitamin K (μg), vitamin B1 (mg), vitamin B2 (mg), niacin (mg), vitamin B6 (mg), vitamin B12 (μg), folic acid (μg), pantothenic acid (mg), biotin (μg), vitamin C (mg), sodium (g), potassium (mg), calcium (mg), magnesium (mg), phosphorus (mg), iron (mg), zinc (mg), copper (mg), manganese (mg), iodine (μg), selenium (μg), chromium (μg), and molybdenum (μg). In addition, alcohol (g) and caffeine (mg) intakes were investigated, as suggested risk factors for SB [19]. Body mass index (kg/m^2^) was calculated based on self-reported height and weight data from the FFQg.

### 2.7. Statistical Analysis

We used SPSS version 26 (IBM, Tokyo, Japan) for statistical analyses. Values of *p* < 0.05 were considered to indicate significant associations. The chi-squared test or Welch’s *t* test was used to investigate significant differences between the SB and NSB groups.

To identify nutrients related with SB, odds ratios and 95% confidence intervals (CIs) were calculated using a backward stepwise logistic regression model. Variables showing values of *p* < 0.2 on the chi-squared test or Welch’s *t* test were selected for inclusion in the model. Model fit was assessed using the Hosmer–Lemeshow test.

As a subgroup analysis, we selected students showing intakes in the top and bottom quartiles of nutrients appearing related to SB. Differences in nutrient intakes were compared between students with and without SB.

## 3. Results

**Figure 2:**
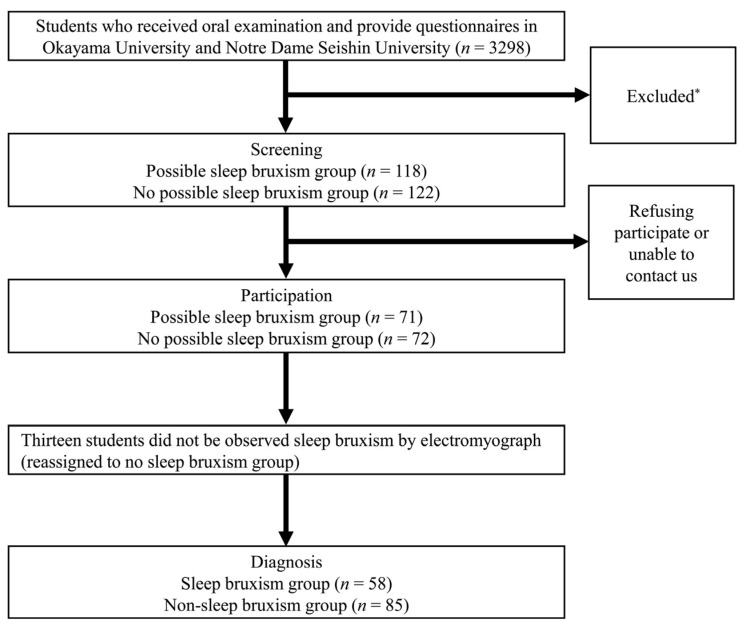
Process of assignment to the sleep bruxism and non-sleep bruxism groups. * Students who answered “yes” to Q1 and “frequently/sometimes/rarely” to Q2 or Q3 or had abnormal tooth wear were excluded.

Figure 2 shows a flowchart for the process of assignment to the SB and NSB groups (Figure 2). A total of 3298 students underwent oral examination and completed the questionnaires. We then assigned 118 students to the possible SB group and 122 students to the non-possible SB group after screening based on the process in Figure 1. After excluding students who refused to participate in the present study or were unable to contact us, we clarified the status of 143 participants (71 and 72 students as possible SB and NSB groups, respectively). Thirteen students did not exhibit SB according to EMG and were reassigned to the NSB group. Finally, we enrolled 58 students in the SB group and 85 students in the NSB group.

Differences in sex, age, systemic disease, medicines being taken regularly, smoking status, and nutrient status between the SB and NSB groups are shown in Table 1. Ten items (sex, age, height, weight, alcohol, dietary fiber, folic acid, vitamin C, sodium, and chromium) showed values of *p* < 0.2.

Table 2 shows the results of the logistic regression analysis. The final model included sex, age, and dietary fiber. Age appeared possibly related with SB (odds ratio, 1.32; *p* = 0.005; 95%CI, 1.08–1.60), but sex (odds ratio, 2.11 (ref. female); *p* = 0.058, 95%CI, 0.97–4.58) and dietary fiber (odds ratio, 0.91; *p* = 0.059; 95%CI, 0.83–1.00) were not. The model fit parameter was good (Hosmer–Lemeshow test, *p* = 0.603).

The upper and lower quartiles of dietary fiber intake were ≥13.2 g and ≤8.7 g. We selected 74 students (36 students from the SB group and 38 students from the NSB group). A subgroup analysis showed that students with SB took significantly less dietary fiber than those without SB (SB group, 10.4 ± 4.6 g; NSB group, 13.4 ± 6.1 g; *p* = 0.022).

## 4. Discussion

To the best our knowledge, the present study represents the first exploratory research to suggest an association between SB and nutrients. Sex, age, height, weight, alcohol, dietary fiber, folic acid, vitamin C, sodium, and chromium all tended to be associated with SB (*p* < 0.2). Logistic modeling showed that SB was significantly associated with age (odds ratio, 1.32; *p* = 0.005; 95%CI, 1.09–1.60) and tended to be associated with sex (odds ratio, 2.11 (ref. female); *p* = 0.058, 95%CI, 0.97–4.58) and dietary fiber (odds ratio, 0.91; *p* = 0.059; 95%CI, 0.83–1.00). The subgroup analysis showed that students with SB had a lower intake of dietary fiber than those without SB (SB group, 10.4 ± 4.6 g; NSB group, 13.4 ± 6.1 g; *p* = 0.022). Intake of dietary fiber might thus influence SB.

According to the National Health and Nutrition Survey Japan 2018, the median intake of dietary fiber among 20- to 29-year-olds was 11.3 g in males and 10.7 g in females. In the present study, students with SB showed a lower intake of dietary fiber (medians: males, 9.9 g; females, 9.8 g) than students with no SB (medians: male, 11.7 g; female, 10.8 g). This trend was clearer in the subgroup analysis (SB group, 10.4 ± 4.6 g; NSB group, 13.4 ± 6.1 g; *p* = 0.022). Our result suggests that a lower intake of dietary fiber is related to SB. SB occurs after micro-arousal during NREM stages 1 and 2 [5,6]. St-Onge et al. reported that higher intake of dietary fiber predicted less stage 1 and more SWS, indicating NREM stage 3 and 4 [26]. More dietary fiber might decrease SB via the promotion of deep sleep.

Dietary fiber may improve sleep disorders from the perspective of the brain–gut axis or brain–gut–microbiota axis [27]. Dietary fiber can be divided into soluble and insoluble types. The metabolism of soluble dietary fiber in the colon increases the type and quantity of probiotics and their representative metabolites (e.g., short-chain fatty acids (SCFAs)) [28]. SCFAs are mainly produced by Bifidobacterium, Lactobacillus, Rosetella, Bacteroidetes, and Firmicutes spp. [27]. SCFAs promote the secretion of serotonin (5-hydroxytryptamine) from the enterochromaffin cells, increasing the level of sleep aids in the body [27]. Insoluble dietary fiber then increases the abundance of Bifidobacteriales and Lactobacillales spp. [29]. This suggests that insoluble dietary fiber indirectly increases the amount of SCFA. Dietary fiber might therefore deepen sleep stage and thus improve SB. We further analyzed the relationships between SB and soluble dietary fiber (odds ratio, 0.663; *p* = 0.063; 95%CI, 0.430–1.023) and between SB and insoluble dietary fiber (odds ratio, 0.869; *p* = 0.046; 95%CI, 0.757–0.997). These results support the notion that both soluble and insoluble dietary fibers have the potential to decrease SB. Wang et al. reported on the microbiota–gut–brain axis in sleep disorders and suggested that manipulating gut microbiota might represent a promising avenue for the development of novel interventions for sleep disorders [30]. The intake of dietary fiber might thus influence SB through the brain–gut–microbiota axis.

Age may be a risk factor for SB in Japanese young adults. Kato et al. indicated that self-reported SB correlates with age [31]. The prevalence of self-reported SB in Japan is 8.2% among those 18–29 years of age, 11.5% among those 30–39 years of age, 10.5% among those 40–49 years of age, 50–59% years old, and 4.6% among those >60 years of age. This result showed the possibility that the prevalence of SB increases until 40–49 years of age. Strausz et al. conducted a follow-up of young Finns (initially aged 14 years) over a 9-year period and found that self-reported SB increased from 13.7% at baseline to 21.7% at follow-up [32]. Wetselaar et al. reported that the odds of having SB among Dutch adolescents was 2.3-times higher at 23 years old than at 17 years old [33]. These studies suggest that the prevalence of SB increases as young adults age. As the present study included participants at 18–32 years old, our results suggested age as a risk factor for SB in Japanese young adults.

Whether sex is related to SB remains unclear. In the present study, students reporting SB that had been pointed out by family members was more common among females (10.3%) than males (7.5%; *p* = 0.004, data not shown). Wetselaar et al. reported that females showed a higher prevalence of SB than males [33,34]. Pontes and Prietsch [35] reported that Brazilian females over 18 years of age tended to show a higher odds ratio for SB than males (odds ratio, 1.17; *p* = 0.378; 95%CI, 0.81–1.68). However, Cavallo et al. [36] found no sex difference for SB among Italian university students. Smardz et al. [37] reported that males exhibited more severe SB than females. Macek et al. [38] reported male sex as an independent risk factor for phasic SB. Guo et al. [39] reported male sex as a risk factor for SB among children 0–12 years of age. Wetselaar et al., Pontes and Prietsch, and Cavallo et al. assessed SB using questionnaires, while Smardz et al. and Macek et al. diagnosed SB using polysomnography (PSG). The gold standard for diagnosing SB is PSG [23]. Such variabilities might have been caused by differences in the determination of SB (questionnaire vs. PSG) and subject age.

The key strength of the present study was that we used electromyography to diagnose SB. Some studies have used questionnaires based on the International Classification of Sleep Disorders—Third Edition (ICSD-3) to investigation SB [40,41,42]. However, using questionnaires alone to diagnose SB is insufficient, because the concordance between the ICSD-3 and PSG diagnostic criteria is only moderate, with an area under the curve of 0.55–0.75 [43]. Conversely, EMG devices show a high level of concordance with PSG (sensitivity, 0.720–1; specificity, 0.916–1) [44], suggesting that EMG devices may allow diagnosis of SB at a similar level to PSG.

Some limitations need to be kept in mind when interpreting our results. First, some selection bias might have been present. All participants belonging to the Department of Food and Nutrition at Notre Dame Seishin University were female, which might have affected the present findings. The intake of nutrients did not differ significantly between females from Okayama University and Notre Dame Seishin University except for alcohol, vitamin K, and iodine (data not shown). Second, we did not investigate single nucleotide polymorphisms (SNPs) related to SB. Some SNPs (rs2736100, SNP rs6313, rs2770304, rs4941573, and rs2770304) have been reported as risk factors for SB [38,45]. Third, since participants who belonged to the NSB group did not undergo electromyographic observation for SB, we did not compare differences in the numbers of episodes and bursts. Forth, all variables included in the logistic regression model (*p* < 0.2) were within the standard deviation. It is necessary to pay attention to interpreting the association between sleep bruxism and nutrients. It is unclear whether small differences in nutrition intake influence sleep bruxism. The effect of nutrients needs to be verified in an interventional trial.

In conclusion, dietary fiber intake appeared to be associated with SB. We hypothesized that increasing dietary fiber intake would improve SB in young adults. To clarify the effect of dietary fiber intake on SB, an interventional trial needs to be performed as confirmatory (evidential) research.

## Figures and Tables

**Table 1:** Differences in parameters between the sleep bruxism and non-sleep bruxism groups.

		SB Group	NSB Group	*p*
		*n* = 58	*n* = 85	
Sex	Male	24 (41.4) ^*^	20 (23.5)	0.023 ^†^
Underlying systemic disease	Yes	6 (10.3)	5 (5.9)	0.353
Medicines taken regularly	Yes	17 (29.3)	21 (24.7)	0.567
Smoking status	Current	1 (1.7)	2 (2.4)	0.355
	Past	3 (5.2)	1 (1.2)	
Age		21.9 ± 2.5 ^‡^	20.7 ± 1.6	0.002 ^§^
PSQI score		5.2 ± 2.1	5.0 ± 1.9	0.560
Height		163.7 ± 8.7	160.8 ± 8.0	0.039
Weight		55.3 ± 8.5	52.6 ± 8.3	0.068
BMI		20.5 ± 1.7	20.3 ± 2.1	0.492
Nutrients				
Alcohol (g)		4.9 ± 8.2	2.0 ± 4.2	0.014
Biotin (μg)		29.5 ± 11.3	29.7 ± 8.5	0.908
Caffeine (mg)		1.4 ± 2.7	2.1 ± 3.1	0.219
Calcium (mg)		448.0 ± 230.8	445.9 ± 162.6	0.947
Carbohydrate (g)		236.5 ± 90.9	237.6 ± 79.2	0.938
Cholesterol (mg)		352.7 ± 162.1	331.6 ± 108.2	0.387
Chromium (μg)		6.8 ± 2.2	7.4 ± 2.2	0.123
Copper (mg)		1.0 ± 0.3	1.0 ± 0.3	0.319
Dietary fiber (g)		10.6 ± 3.7	11.9 ± 4.3	0.056
Energy (kcal)		1872.7 ± 569.2	1852.3 ± 479.0	0.818
Folic acid (μg)		221.0 ± 92.7	243.3 ± 105.0	0.193
Iodine (μg)		590.4 ± 560.0	626.8 ± 626.3	0.722
Iron (mg)		6.3 ± 2.1	6.8 ± 1.9	0.217
Lipid (g)		66.5 ± 19.8	65.9 ± 17.4	0.851
Magnesium (mg)		208.4 ± 69.2	218.4 ± 60.0	0.359
Manganese (mg)		2.3 ± 0.9	2.5 ± 0.7	0.417
Molybdenum (μg)		157.9 ± 76.4	157.8 ± 55.1	0.997
Monounsaturated fatty acid (g)		24.0 ± 6.9	23.9 ± 6.5	0.88
n-3 Polyunsaturated fatty acid (g)		2.0 ± 0.8	1.9 ± 0.6	0.909
n-6 Polyunsaturated fatty acid (g)		11.3 ± 3.7	11.3 ± 3.4	0.995
Niacin equivalents (mg)		27.0 ± 8.0	27.5 ± 7.1	0.675
Pantothenic acid (mg)		5.4 ± 1.7	5.4 ± 1.3	0.952
Phosphorus (mg)		933.9 ± 301.2	931.7 ± 218.9	0.959
Polyunsaturated fatty acid(g)		13.3 ± 3.9	13.3 ± 3.9	0.988
Potassium (mg)		1967.7 ± 657.8	2076.8 ± 626.5	0.318
Protein (g)		65.9 ± 17.4	67.0 ± 16.9	0.733
Retinol activity equivalents (μg)		371.9 ± 234.3	407.9 ± 247.3	0.384
Saturated fatty acid (g)		21.8 ± 7.8	21.4 ± 6.3	0.699
Selenium (μg)		60.1 ± 17.2	60.9 ± 15.1	0.775
Sodium (g)		7.5 ± 2.7	8.1 ± 2.7	0.167
Vitamin B1 (mg)		1.0 ± 0.3	1.1 ± 0.3	0.414
Vitamin B12 (μg)		3.8 ± 2.0	3.8 ± 1.6	0.938
Vitamin B2 (mg)		1.1 ± 0.4	1.1 ± 0.3	0.967
Vitamin B6 (mg)		1.0 ± 0.3	1.1 ± 0.3	0.487
Vitamin C (mg)		58.1 ± 28.9	66.4 ± 34.6	0.137
Vitamin D (μg)		3.5 ± 2.0	3.5 ± 1.6	0.903
Vitamin K (μg)		186.1 ± 99.7	204.4 ± 114.5	0.322
Water (g)		845.7 ± 298.1	815.3 ± 244.4	0.505
Zinc (mg)		7.9 ± 2.5	7.9 ± 2.0	0.901
α-Tocopherol (mg)		6.2 ± 2.0	6.5 ± 2.0	0.379

^*^ *n* (%); ^†^ Chi-squared test or Fisher exact test; ^‡^ Mean ± SD; ^§^ Student’s *t* test or Welch’s *t* test. SB, sleep bruxism; NSB, non-sleep bruxism; PSQI, Pittsburgh sleep quality index; BMI, body mass index.

**Table 2:** Odds ratio of sleep bruxism.

		Odds Ratio	*p*	95%CI
Sex	Female	Ref.		
	Male	2.11	0.058	0.97–4.58
Age		1.32	0.005	1.09–1.60
Dietary fiber		0.91	0.059	0.83–1.00

Ref., reference group; CI, confidence interval. A backward stepwise selection method was applied, including sex, age, height, weight, alcohol, dietary fiber, folic acid, vitamin C, sodium, and chromium.

## Data Availability

The data presented in this study are available on request from the corresponding author. The data are not publicly available, due to ethical issues.

## Supplementary Materials

The following supporting information can be downloaded at: https://www.mdpi.com/article/10.3390/jcm12072623/s1, Supplementary File S1: Questionnaire.
