# Supplementary material for: Nutrients Associated with Sleep Bruxism
Source: J Clin Med. 2023 Mar 31;12(7):2623. doi: 10.3390/jcm12072623 (PMC10095372; doi:10.3390/jcm12072623)
Supplement: Supplementary file 1 [file jcm-12-02623-s001.zip › jcm-2263031-supplementary.pdf]

Questionnaire

Name (   )

Student number (   )

Sex (male / female)

Age (   )

1. Within the last 3 months, has anyone told you that you grind your teeth while sleeping?

( ) No ( ) Yes

2. Within the last 3 months, have you ever had tired jaw muscles when you wake up in the morning?

( ) No ( ) Rarely ( ) Sometimes ( ) Frequently

3. Within the last 3 months, have you had temporary headaches when you wake up in the morning?

( ) No ( ) Rarely ( ) Sometimes ( ) Frequently

4. Within the last 3 months, do you have difficulty opening your mouth when you wake up in the morning?

( ) Never ( ) Rarely ( ) Sometimes ( ) Frequently

5. It is said that there is a relationship between systemic diseases and oral health. Do you have any systemic illnesses?

( ) No

( ) Yes ⇒ Name of disease ( )

6. Have you taken any hospital medication in the last 3 months?

( ) No

( ) Yes ⇒Name of medicine ( )

7. Do you smoke cigarettes?

( ) Never ( ) Past smoking ( ) Yes (currently smoking)

Instructions: The following questions relate to your usual sleep habits during the past month only. Your answers should indicate the most accurate reply for the majority of days and nights in the past month. Please answer all questions.

8. During the past month, what time have you usually gone to bed at night? __________________

9. During the past month, how long (in minutes) has it usually take you to fall asleep each night? __________

10. During the past month, what time have you usually gotten up in the morning? ___________________

11. During the past month, how many hours of actual sleep did you get at night?
(This may be different than the number of hours you spent in bed.) ___________________

12. During the past month, how often have you had trouble sleeping because…
a. Cannot get to sleep within 30 minutes
Not during the past month ( ); Less than once a week ( );
Once or twice a week ( ); Three or more times a week ( )

b. Wake up in the middle of the night or early morning
Not during the past month ( ); Less than once a week ( );
Once or twice a week ( ); Three or more times a week ( )

c. Have to get up to use the bathroom
Not during the past month ( ); Less than once a week ( );
Once or twice a week ( ); Three or more times a week ( )

d. Cannot breathe comfortably
Not during the past month ( ); Less than once a week ( );
Once or twice a week ( ); Three or more times a week ( )

e. Cough or snore loudly
Not during the past month ( ); Less than once a week ( );
Once or twice a week ( ); Three or more times a week ( )

f. Feel too cold
Not during the past month ( ); Less than once a week ( );
Once or twice a week ( ); Three or more times a week ( )

g. Feel too hot
Not during the past month ( ); Less than once a week ( );
Once or twice a week ( ); Three or more times a week ( )

h. Have bad dreams
Not during the past month ( ); Less than once a week ( );
Once or twice a week ( ); Three or more times a week ( )

i. Have pain
Not during the past month ( ); Less than once a week ( );

Once or twice a week ( ); Three or more times a week ( )
j. Other reason(s), please describe:
Not during the past month ( ); Less than once a week ( );
Once or twice a week ( ); Three or more times a week ( )

13. During the past month, how often have you taken medicine to help you sleep (prescribed or "over the counter")?
Not during the past month ( ); Less than once a week ( );
Once or twice a week ( ); Three or more times a week ( )

14. During the past month, how often have you had trouble staying awake while driving, eating meals, or engaging in social activity?
Not during the past month ( ); Less than once a week ( );
Once or twice a week ( ); Three or more times a week ( )

15. During the past month, how much of a problem has it been for you to keep up enough enthusiasm to get things done?
No problem at all ( ); Only a very slight problem ( );
Somewhat of a problem ( ); A very big problem ( )

16. During the past month, how would you rate your sleep quality overall?
Very good ( ); Fairly good ( ); Fairly bad ( ); Very bad ( )

17. Do you have a bed partner or roommate?
No bed partner or roommate ( ); Partner/roommate in other room ( );
Partner in same room but not same bed ( ); Partner in same bed ( )

If you have a roommate or bed partner, ask him/her how often in the past month you have had:
a. Loud snoring
Not during the past month ( ); Less than once a week ( );
Once or twice a week ( ); Three or more times a week ( )

b. Long pauses between breaths while asleep
Not during the past month ( ); Less than once a week ( );
Once or twice a week ( ); Three or more times a week ( )

c. Legs twitching or jerking while you sleep
Not during the past month ( ); Less than once a week ( );
Once or twice a week ( ); Three or more times a week ( )

d. Episodes of disorientation or confusion during sleep

Not during the past month ( ); Less than once a week ( );

Once or twice a week ( ); Three or more times a week ( )

e. Other restlessness while you sleep, please describe:

Not during the past month ( ); Less than once a week ( );

Once or twice a week ( ); Three or more times a week ( )
